# CT-Based Radiomic Signatures Associated with Serum CEA Status in Colon Cancer

**DOI:** 10.3390/diagnostics16081221

**Published:** 2026-04-19

**Authors:** Demet Doğan, Coşku Öksüz, Özgür Çakır, Oğuzhan Urhan

**Affiliations:** 1 Department of Radiology, Faculty of Medicine, İstanbul Okan University, 34947 İstanbul, Türkiye; 2 Department of Electrical and Electronics Engineering, Izmir Bakircay University, 35665 Izmir, Türkiye; cosku.oksuz@bakircay.edu.tr; 3 Department of Radiology, Faculty of Medicine, Kocaeli University, 41001 Kocaeli, Türkiye; cakirozgur@hotmail.com; 4 Department of Electronics & Telecommunication Engineering, Kocaeli University, 41001 Kocaeli, Türkiye; urhano@kocaeli.edu.tr

**Keywords:** colonic neoplasms, carcinoembryonic antigen, radiomics, tomography, machine learning

## Abstract

**Background/Objectives:** Carcinoembryonic antigen (CEA) is widely used in colon cancer management; however, its diagnostic and prognostic accuracy is limited by biological variability, as well as false-positive or false-negative results. Radiomics provides quantitative descriptors of tumor heterogeneity and offers objective assessment of tumor characteristics. This study aimed to evaluate the potential of computed tomography (CT)-based radiomic features to distinguish between CEA-positive and CEA-negative colon cancer patients. **Methods:** In this retrospective study, 150 patients with histopathologically confirmed colon cancer were screened, and 109 were eligible after image-quality assessment (53 CEA-positive, 56 CEA-negative). A total of 107 radiomic features were extracted from preoperative contrast-enhanced CT images. After z-score normalization, feature robustness was assessed using intra- and inter-observer agreement. Correlation-based feature selection (|ρ| ≥ 0.7) was applied. Five machine-learning classifiers—Support Vector Machine (SVM), Decision Tree, Ensemble, k-Nearest Neighbor (k-NN), and Neural Network (NN)—were trained using stratified 5-fold cross-validation. Performance was evaluated using accuracy, recall, specificity, F1-score, and ROC-AUC. **Results:** The best performance was obtained with 41 selected features. The k-NN classifier achieved the highest accuracy (77.4 ± 2%) and ROC-AUC (0.8523 ± 0.013), while SVM and NN achieved the highest recall (83.0 ± 0.3). These models showed balanced and robust performance in distinguishing CEA-positive from CEA-negative patients. **Conclusions:** CT-based radiomic analysis combined with machine learning—particularly k-NN, SVM, and neural network classifiers—showed promising performance in differentiating colon cancer patients according to serum CEA status. Radiomic features may provide imaging-based information associated with serum biomarkers such as CEA, potentially enhancing tumor characterization and supporting more personalized decision-making.

## 1. Introduction

Colorectal cancer is one of the most common malignancies worldwide and represents a significant public health concern. According to the International Agency for Research on Cancer (IARC) and the World Health Organization (WHO), colorectal cancer is the third most frequently diagnosed cancer globally and the second leading cause of cancer-related deaths [1,2,3]. Due to this substantial disease burden, both early detection and the identification of reliable prognostic biomarkers and imaging-based parameters carry critical importance.

Carcinoembryonic antigen (CEA) is a glycoprotein found in the blood and remains one of the most widely used and easily accessible biomarkers in the management of colorectal cancer [4]. In healthy, non-smoking adults, serum CEA levels below 3.0 ng/mL are considered normal, whereas in smokers, due to higher baseline levels, the upper limit may reach 5 ng/mL. Therefore, in clinical practice, 5 ng/mL is commonly used as the threshold for distinguishing normal from elevated CEA levels. Pretreatment CEA levels between 5 and 10 ng/mL are generally associated with localized disease and a favorable prognosis, while levels exceeding 10 ng/mL indicate a higher risk of recurrence and poorer outcomes [5]. Serum CEA levels provide valuable clinical information, particularly for postoperative surveillance and prognostic evaluation, and are routinely recommended in international guidelines [6,7,8,9]. However, CEA levels are not always directly correlated with tumor burden or biological aggressiveness. In metastatic colorectal cancer, CEA levels are often elevated; however, in some advanced-stage cases, they may remain within normal limits [10,11,12]. Therefore, biological variability may lead to false-negative or false-positive results. Thus, although clinically useful, CEA alone is insufficient for reliable diagnostic and prognostic decision-making, highlighting the need for complementary tools that can better reflect tumor biology.

Diagnostic imaging undergoes a transformative shift from a discipline based solely on visual interpretation to a data-driven science, integrating quantitative information extracted from images into clinical decision-making. Medical images are complex data sources that reflect the physical and biological properties of tissues. With advances in computational technologies and artificial intelligence algorithms, these images can now be systematically analyzed and decomposed into hundreds of quantitative features—such as intensity, shape, and texture—enabling the objective assessment of tumor heterogeneity. This entire process, known as radiomics, is transforming radiology into a predictive, data-oriented discipline. Radiomics has emerged as a significant field of research in personalized oncology, offering a non-invasive means to characterize the microstructural and biological properties of tumors [13]. These quantitative features are thought to reflect underlying molecular and pathological characteristics, such as tumor heterogeneity, angiogenesis, and gene expression, providing valuable insights beyond visual assessment.

Computed tomography (CT)-based radiomic features can quantitatively characterize tumor microstructure and have been linked to clinical outcomes, treatment response, and prognostic indicators. Previous studies have demonstrated that radiomic features can predict survival, risk of metastasis, and treatment response in colorectal cancer [14,15,16]. Furthermore, radiomic features may provide complementary imaging-based information related to serum biomarkers such as CEA and may contribute to improved tumor characterization. To the best of our knowledge, the relationship between CT-based radiomic features and serum CEA status has not been sufficiently investigated in the literature. Therefore, this study aims to explore whether radiomic features may provide imaging-based information associated with CEA levels in colon cancer patients. In this context, this study aimed to evaluated the role of radiomic features derived from preoperative CT in distinguishing CEA-positive from CEA-negative colon cancer patients and their potential contribution to clinical decision-making.

## 2. Materials and Methods

### 2.1. Study Population

This study included 150 adult patients (75 women and 75 men) with histopathologically confirmed colon cancer who underwent preoperative abdominal CT imaging at our institution between January 2021 and October 2025 and had untreated serum CEA measurements available. A total of 41 patients were excluded due to poor image quality, the absence of preoperative CEA values, or incomplete clinical records. The final cohort consisted of 109 eligible patients (53 CEA-positive and 56 CEA-negative). All abdominal CT scans were reviewed to ensure adequate image quality and complete visualization of the colon. Patients with poor image quality or incomplete clinical and laboratory data were excluded. Radiomic features were extracted from segmented tumor regions on preoperative CT images. Serum CEA measurements were obtained within 10 days prior to the CT examination to ensure temporal consistency between imaging and laboratory data. Patients with CEA levels of 5 ng/mL or higher were classified as CEA-positive, while those with CEA levels below 5 ng/mL were classified as CEA-negative. The extracted features were then compared between the CEA-positive and CEA-negative groups to evaluate their discriminative potential.

### 2.2. Radiological Protocol

All abdominal CT examinations were performed using a 64-slice CT scanner (Optima CT 660, General Electric Medical Systems, Milwaukee, WI, USA). Patients were scanned in the supine position. The protocol included spiral scanning with a tube voltage of 120 kV and a tube current of 70–120 mAs. Slice thicknesses were set to 1.25, 2.5, and 5 mm, and multiplanar reconstructions were obtained. All examinations were contrast-enhanced, and a water-soluble, non-ionic, high-iodine concentration contrast agent (300–350 mg/mL) was administered intravenously via an 18-gauge catheter placed in the antecubital vein using an automatic dual-injector system at a dose of 1 mL/kg (90–100 mL at a rate of 3–5 mL/s. Imaging was initiated 65 s after the delay, and all evaluations were performed on images obtained in the portal venous phase.

### 2.3. Radiomic Feature Extraction, Reliability Analysis, and Modeling

A total of 107 quantitative radiomic features (including first-order statistics, shape, and texture features derived from GLCM, GLRLM, etc.) were extracted from the manually segmented tumor regions on the contrast-enhanced CT images. Tumor segmentation was performed manually by an experienced abdominal radiologist with 13 years of dedicated experience. Tumor segmentation was performed using the open-source software 3D Slicer (version 5.8.1), and radiomic features were subsequently extracted using the PyRadiomics library (version 3.1.0). To ensure the robustness and reproducibility of the extracted radiomic features, both intra- and inter-observer reliability analyses were performed on a randomly selected subset of 30 patients. One experienced radiologist performed the tumor segmentation twice with a 2-week interval (intra-observer), and a second experienced radiologist performed the segmentation once (inter-observer). The Intra-class Correlation Coefficient (ICC) was calculated for all 107 features. Features with an ICC value of less than 0.8 for intra- or inter-observer agreement were excluded from subsequent analysis, as they were deemed unreliable.

The remaining robust features underwent Z-score normalization, followed by correlation-based feature selection using a threshold of |ρ| ≥ 0.8, resulting in a final set of 41 features. This feature selection step was implemented as a preliminary unsupervised filtering procedure prior to model training. Since the method is based on feature–feature correlations and does not utilize class labels, it does not directly exploit discriminative information between classes, thereby reducing the risk of label-driven information leakage. The primary objective of this step was to eliminate redundant features and stabilize the feature space rather than to optimize predictive performance.

Then, machine learning models (SVM, Decision Tree, Ensemble, k-NN, NN) were trained using stratified 5-fold cross-validation. The models were evaluated strictly using the test folds to ensure unbiased performance estimation. Hyperparameter optimization for each model was performed using Bayesian Optimization over 30 iterations, followed by refinement with Grid Search, on the training folds.

### 2.4. Statistical Analysis

Radiomic features extracted from CT images were scaled using z-score normalization after excluding missing or non-diagnostic data. To reduce multicollinearity, Pearson correlation coefficients were calculated, and in highly correlated pairs (|ρ| ≥ 0.6–0.8), the feature with lower variance was removed.

Machine learning models, including SVM, Decision Tree, Ensemble Learning, k-NN, and NN, were applied. Hyperparameter optimization was performed using Bayesian optimization, followed by grid search refinement.

Model performance was evaluated using stratified 5-fold cross-validation to ensure generalizability. Evaluation metrics included Accuracy, Recall (Sensitivity), Specificity, Precision, F1 score, and ROC-AUC. For each metric, mean values and 95% confidence intervals were calculated from the cross-validation results. Special emphasis was placed on Recall and ROC-AUC, given their clinical importance for correctly identifying CEA-positive cases and reducing false negatives.

### 2.5. Dataset

In this study, radiomic features extracted from CT images of patients with colon tumors were utilized. The images belonged to clinically confirmed CEA-positive and CEA-negative patient groups. For each patient, radiomic features obtained from different cross-sectional levels were provided as separate Excel files. All files were merged to create a patient-specific primary database. Initially, the dataset contained 107 features, with each row representing a feature vector derived from CT images. Tumor segmentation was performed manually by an experienced abdominal radiologist. For each patient, images were obtained from three slices: the one with the largest tumor area (central slice) and the immediately adjacent cranial and caudal slices. The images were saved in TIFF format. The segmented tumor images were subsequently used for radiomic feature extraction and preprocessing steps.

### 2.6. Data Preprocessing

First, missing values and non-diagnostic records were excluded from the dataset. All features were scaled using z-score normalization. Each feature was labeled using the structure “ImageType_FeatureClass_FeatureName,” enabling class-level correlation analysis. The dataset was divided into two classes: CEA-positive (1) and CEA-negative (0).

### 2.7. Feature Selection

To reduce multicollinearity among features, correlation-based feature pruning was applied [17,18]. Pearson correlation coefficients were calculated for all feature pairs, and pairs with |ρ| ≥ threshold were identified as highly correlated. For each correlated pair, the feature with lower variance was removed. This process was repeated for correlation thresholds of 0.6, 0.7, and 0.8.

The resulting feature dimensions were as follows:

- One hundred and seven features: Original dataset without reduction.
- Forty-one features: Final selected feature set at correlation threshold 0.8.
- Thirty-one and twenty-two features: Reduced sets obtained under stricter thresholds (0.7 and 0.6).

### 2.8. Classification

Five machine learning classifiers were evaluated on the selected feature sets: SVM, Decision Tree, Ensemble Learning, k-NN, and NN. Hyperparameters for each classifier were optimized using Bayesian optimization over 30 iterations [19], which probabilistically models the parameter space to identify the best configuration with fewer trials.

All models were evaluated using stratified 5-fold cross-validation (CV-5), ensuring reliable generalization testing despite the limited sample size. Hyperparameters were further refined via grid search. Each model was trained and tested using CV-5, providing a statistically reliable assessment of generalization.

### 2.9. Performance Evaluation

The performance of the models was assessed using Accuracy, Recall (sensitivity), Specificity, F1 score, Precision, and Brier score, as defined in Equations (1)–(6). In Equation (6), the term $p_{i}$ represents the model’s predicted probability for the positive class, $y_{i}$ indicates the true binary outcome, and N corresponds to the sample size used in the evaluation. ROC-AUC score was also included in comparisons. For each metric, mean values and ±95% confidence intervals were calculated from the stratified 5-fold cross-validation results. Particular emphasis was placed on Recall and ROC-AUC metrics, as they hold specific clinical importance for correctly identifying CEA-positive patients.

(1) $$Accuracy=\frac{TP+TN}{TP+TN+FP+FN}$$

(2) $$Recall=\frac{TP}{TP+FN}$$

(3) $$Specificity=\frac{TN}{TN+FP}$$

(4) $$F1\;Score=\frac{2.Recall\cdot Precision}{Recall+Precision}$$

(5) $$Precision=\frac{TP}{TP+FP}$$

(6) $$Brier\;Score=\frac{1}{N}\sum_{i=1}^{N}{\left(p_{i}-y_{i}\right)}^{2}$$

## 3. Results

The baseline clinical and pathological characteristics of the study population are summarized in Table 1. The cohort included 109 patients with a mean age of 55.09 ± 16.81 years, of whom 64.2% were male. Based on serum CEA levels, 49.5% of patients were classified as CEA-positive. The distribution of TNM classification and tumor stages demonstrated a heterogeneous patient population.

The distribution of smoking status and its association with serum CEA levels are presented in Table 2. A total of 38 patients (34.9%) were smokers, whereas 71 (65.1%) were non-smokers. The mean serum CEA level was significantly higher in smokers compared to non-smokers (20.78 ± 7.31 vs. 4.08 ± 2.56, *p* = 0.001), indicating that smoking status may act as a confounding factor influencing CEA levels.

In multivariate linear regression analysis, smoking remained significantly associated with increased serum CEA levels after adjusting for age, sex, tumor stage, and tumor type (β = 28.589, SE = 8.806, *p* = 0.003). No statistically significant associations were observed for the other variables included in the model (Table 3).

**Figure 1:**
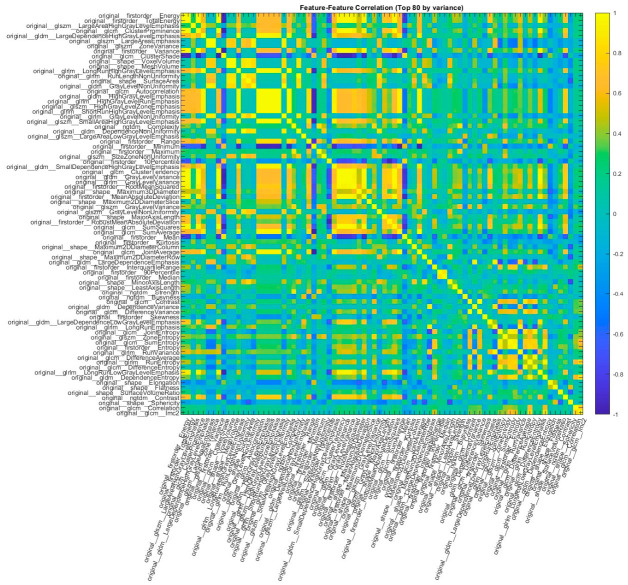
Pairwise correlation matrix of radiomic features in the dataset before dimensionality reduction (top 80 features with the highest variance).

The correlation structure of the radiomic features obtained without dimensionality reduction is shown in Figure 1.

As shown, many features are highly correlated, indicating redundant information in the dataset. Using all 107 features without dimensionality reduction, the classification results are presented in Table 4.

Based on the stratified 5-fold cross-validation results, the k-NN model achieved the highest overall performance with 79.2 ± 2.0% accuracy and 0.8545 ± 0.013 ROC-AUC. This finding indicates that radiomic features exhibit clustering tendencies among similar instances and that neighborhood-based decision mechanisms can successfully capture this structure. The SVM model demonstrated 75.5 ± 2.0% accuracy and 90.6 ± 1.8% specificity, maintaining the lowest false-positive rate. However, its relatively low sensitivity (60.4 ± 2.5%) suggests a tendency to miss some positive cases.

The Ensemble method yielded comparable accuracy (75.5 ± 1.9%) but showed higher sensitivity (67.9 ± 2.3%), providing a more balanced ability to detect positive cases. In contrast, the Decision Tree and NN models exhibited lower accuracy and F1 scores, indicating more limited discriminative power. Overall, when all features were used, models achieved high specificity but lower sensitivity, meaning they were more effective at correctly classifying negative cases while partially failing to detect positive cases.

**Figure 2:**
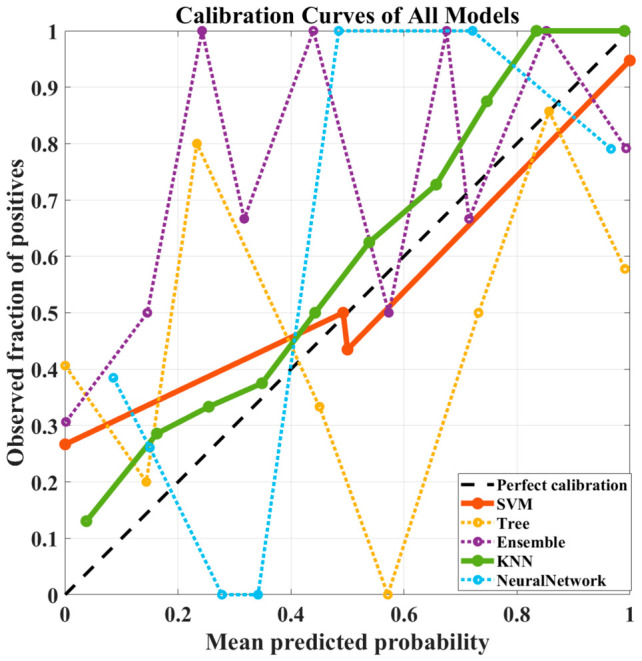
Calibration Curves of All Models Using the Full Radiomic Feature Set (No Feature Selection). The dashed diagonal line indicates perfect calibration.

In addition to discrimination performance, calibration analysis was conducted to assess the reliability of predicted probabilities. As illustrated in Figure 2, calibration curves reveal notable differences among models despite similar classification performance. The k-NN model shows a more consistent alignment with the diagonal reference line, indicating relatively better calibration behavior, particularly at higher probability ranges. In contrast, the Decision Tree and Neural Network models exhibit larger deviations and instability across probability bins, suggesting less reliable probability estimates.

In conclusion, on the dataset without dimensionality reduction, the k-NN and SVM models outperformed the other methods in terms of overall classification performance and stability. Furthermore, the calibration analysis supports these findings by demonstrating that the k-NN model provides more reliable probability estimates, reinforcing its suitability for applications where probabilistic interpretation is critical.

**Figure 3:**
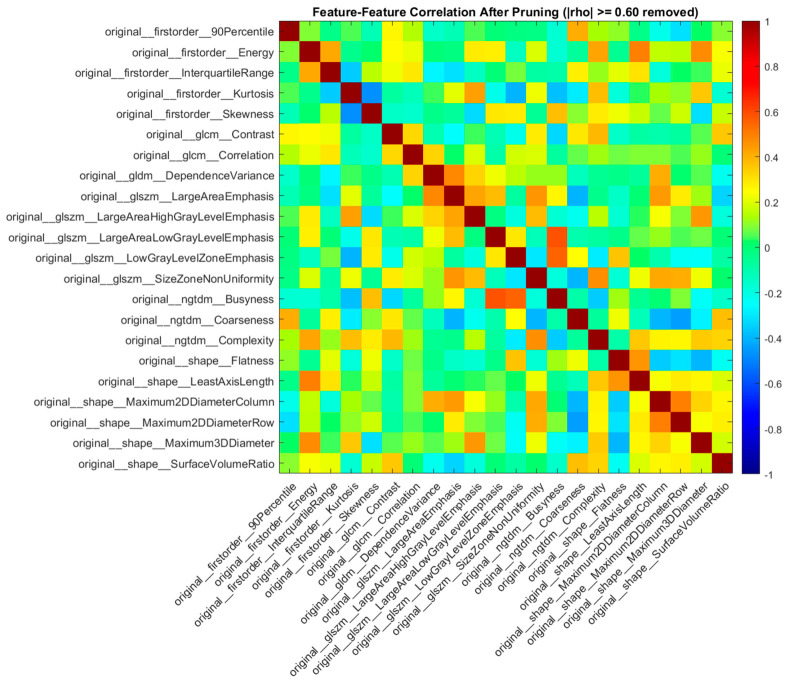
Correlation heatmap of radiomic features after eliminating highly correlated features using a correlation threshold of (|ρ| ≥ 0.6).

After applying correlation thresholding (|ρ| ≥ 0.6) to eliminate highly correlated features, the correlation structure of the new feature set was obtained, and is shown in Figure 3. As seen in Figure 3, the strong linear relationships among features were reduced mainly, thereby minimizing multicollinearity during model training. After selecting 22 features from the original 107 through correlation-based feature selection, the classification results were obtained, and are presented in Table 5. Overall, the models preserved performance stability after dimensionality reduction, with noticeable improvements in some metrics.

The k-NN model achieved the highest overall performance with 80.2 ± 2.1% accuracy, 77.8 ± 2.0% F1 score, and 0.8592 ± 0.013 ROC-AUC, highlighting its effectiveness in modeling similarity relationships in the reduced radiomic space. The Ensemble model achieved balanced performance with 76.4 ± 2.0% accuracy and 71.7 ± 2.4% sensitivity, excelling in capturing positive cases. The SVM model demonstrated 72.6 ± 2.1% accuracy and 84.9 ± 2.0% specificity, indicating a low false-positive rate. However, its sensitivity remained relatively limited (60.4 ± 2.5%).

The NN achieved 79.2 ± 2.0% accuracy and 92.5 ± 1.7% specificity but lower sensitivity, resulting in higher misclassification among positive cases. The Decision Tree model achieved the lowest performance, with 66.0 ± 2.3% accuracy and 0.6333 ± 0.020 ROC-AUC.

In addition to these performance metrics, the Brier scores reported in Table 5 provide a quantitative assessment of calibration performance under the reduced feature setting. The results indicate that the k-NN model achieved the lowest Brier score (0.163 ± 0.034), suggesting the most reliable probability estimates among the evaluated models. The NN model also demonstrated relatively favorable calibration (0.186 ± 0.035), whereas higher Brier scores observed in the Ensemble (0.273 ± 0.102) and Decision Tree (0.241 ± 0.016) models indicate less stable and less reliable probability predictions. Notably, the SVM model exhibited moderate calibration performance (0.216 ± 0.034), aligning with its balanced discrimination characteristics.

These findings indicate that correlation-based feature selection maintains model performance while improving generalizability, particularly for k-NN and Ensemble models. Furthermore, the calibration analysis suggests that feature reduction not only preserves classification performance but also contributes to more stable and reliable probability estimation, especially for the k-NN model.

The classification results with 31 selected features are shown in Table 3. As presented in Table 6, increasing the number of features from 22 to 31 did not significantly improve performance.

The k-NN model again achieved the best performance, with 78.3 ± 2.0% accuracy, 76.3 ± 2.1% F1 score, and 0.7932 ± 0.014 ROC-AUC, demonstrating strong generalizability across low-dimensional radiomic data. The NN achieved comparable results, with 73.6 ± 2.1% accuracy and 0.7935 ± 0.015 ROC-AUC, partially capturing non-linear feature interactions. The SVM achieved low false-positive rates (86.8 ± 1.9% specificity) but limited sensitivity (56.6 ± 2.5%). The Decision Tree model again showed the weakest results (66.0 ± 2.3% accuracy, 0.7255 ± 0.018 ROC-AUC). The Ensemble model did not improve significantly in this configuration, with weak F1 (56.9 ± 2.5%) and ROC-AUC (0.6778 ± 0.019) scores.

In addition to these performance metrics, the Brier scores reported in Table 6 provide insight into calibration performance under the expanded feature setting. The k-NN model maintained the lowest Brier score (0.163 ± 0.023), indicating consistently reliable probability estimates. In contrast, the SVM model exhibited the highest Brier score (0.314 ± 0.063), suggesting reduced calibration quality despite reasonable classification performance. The Tree (0.208 ± 0.069), Ensemble (0.211 ± 0.021), and NN (0.213 ± 0.051) models showed moderate calibration performance, with noticeable variability across folds. These findings indicate that increasing the number of features does not necessarily improve the reliability of predicted probabilities.

These results indicate that low-dimensional feature sets remain sufficient for classification, while adding correlated variables does not provide meaningful improvement in either discrimination or calibration performance.

As shown in Table 7, using 41 features obtained by applying a correlation threshold of 0.8 improved the classification performance compared to the 22- and 31-feature configurations.

The k-NN model achieved 77.4 ± 2.0% accuracy, 77.8 ± 2.0% F1 score, and 0.8523 ± 0.013 ROC-AUC, once again achieving the highest overall performance. The SVM also performed strongly, with 76.4 ± 2.0% accuracy, 83.0 ± 2.3% sensitivity, and 0.8324 ± 0.014 ROC-AUC, showing balanced discriminative ability. The NN demonstrated competitive results (78.3 ± 2.0% accuracy, 79.3 ± 1.9% F1 score). In contrast, the Decision Tree and Ensemble models achieved lower performance, with weaker generalizability.

In addition to discrimination metrics, the Brier scores reported in Table 7 provide a quantitative assessment of calibration performance for this feature configuration. The k-NN model maintained the lowest Brier score (0.188 ± 0.043), indicating relatively reliable probability estimates. In contrast, the Ensemble model exhibited a substantially higher Brier score (0.495 ± 0.022), suggesting poor calibration despite moderate classification performance. The SVM (0.294 ± 0.058) and NN (0.309 ± 0.053) models showed degraded calibration compared to the lower-dimensional settings, while the Decision Tree (0.231 ± 0.091) demonstrated moderate but unstable calibration behavior. These results indicate that increasing the number of features may negatively affect the reliability of predicted probabilities, even when classification metrics improve.

**Figure 4:**
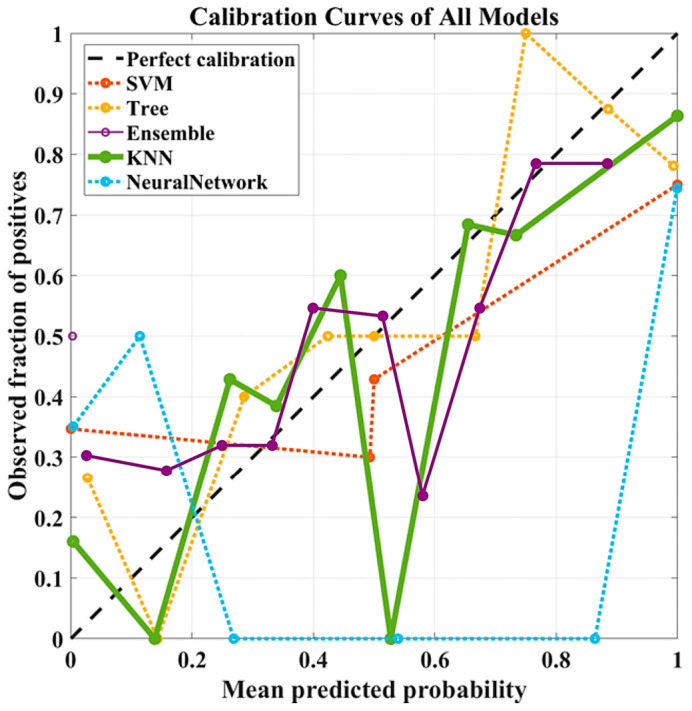
Calibration curves of the evaluated models using 41 radiomic features selected based on a correlation threshold of 0.8. The dashed diagonal line indicates perfect calibration.

As illustrated in Figure 4, calibration curves further support these findings by showing that the k-NN model remains closer to the ideal diagonal line, whereas other models exhibit larger deviations and instability across probability ranges.

These findings indicate that selecting 41 features with a correlation threshold of 0.7 improves classification performance while introducing variability in calibration, highlighting a trade-off between discrimination and probability reliability.

**Figure 5:**
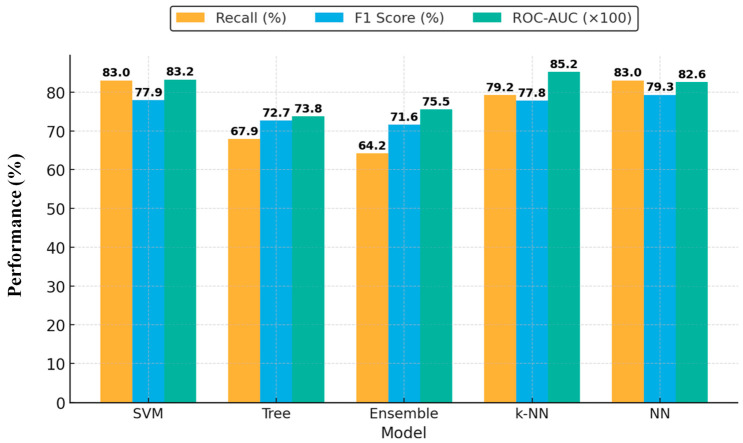
Comparison of performance metrics (Recall, F1 Score, and ROC-AUC) for machine learning models trained with 41 selected radiomic features.

Analysis of classification performance across different feature dimensions demonstrated that models trained with 41 selected features produced the most balanced outcomes. Thus, the scores obtained by the models with 41 features are shown in Figure 5 as a comparative bar plot.

In the 41-dimensional feature space, k-NN, SVM, and NN models performed best in terms of both overall accuracy and discriminative ability (ROC-AUC). The highest recall (83.0 ± 2.3%) was achieved by SVM and NN models, indicating their effectiveness in capturing positive colon cancer cases. The k-NN model also excelled, achieving 77.8 ± 2.0% F1 score and 0.8523 ± 0.013 ROC-AUC, reflecting a strong balance between sensitivity and specificity. These findings show that k-NN and SVM are particularly effective at correctly identifying the positive class (CEA-positive patients), and that high F1 scores demonstrate their ability to balance sensitivity and specificity. Therefore, the dataset with 41 features selected at a correlation threshold of 0.8 enhanced overall model performance, with significant improvements in recall and ROC-AUC metrics, suggesting clinically meaningful advances.

**Figure 6:**
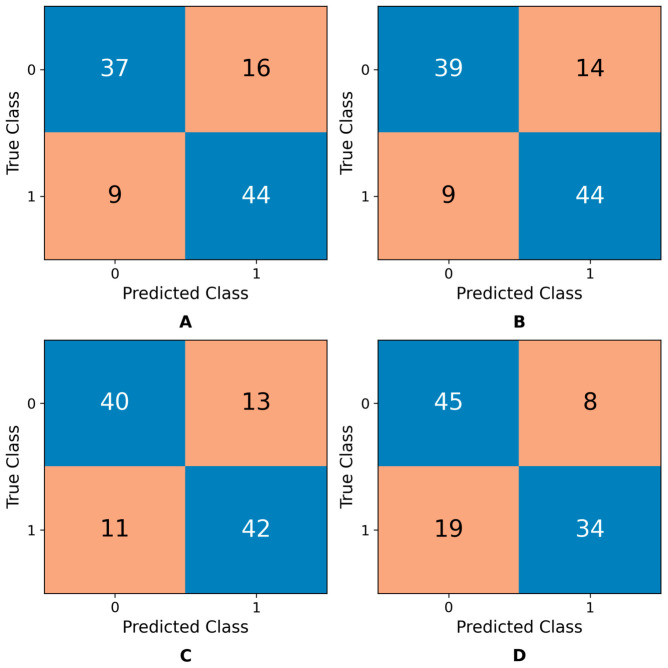
Confusion matrices of (**A**) SVM, (**B**) NN, (**C**) k-NN, and (**D**) Ensemble models for classifying CEA-positive and CEA-negative colon cancer patients. Diagonal cells (blue) represent correctly classified samples, whereas off-diagonal cells indicate misclassifications.

The confusion matrices presented in Figure 6A–D further support these results. Specifically, the SVM and NN models (Figure 6A,B) achieved the highest sensitivity, while the k-NN (Figure 6C) and Ensemble (Figure 6D) models demonstrated balanced and limited performance, respectively.

This emphasizes their ability to capture positive colon cancer cases and reduce false negatives. The k-NN model also demonstrated stable performance, with 42 true positives and 45 true negatives, achieving a strong balance between recall and specificity. In contrast, the Ensemble model misclassified several positive cases as negatives (19 cases), indicating limited generalizability for the positive class. Overall, the combination of confusion matrices and performance metrics demonstrates that the k-NN, SVM, and NN models yield more stable and reliable results in identifying colon cancer cases. Their superior performance in detecting the positive class highlights an important advantage for clinical applications, particularly by reducing false negatives.

### Statistical Comparison of ROC-AUC Using the DeLong Test

The statistical significance of differences in ROC-AUC values between the evaluated models was assessed using the paired DeLong test. The analysis was conducted based on out-of-fold predicted probabilities obtained from stratified 5-fold cross-validation, enabling paired comparisons across models. The AUC values indicate that the k-NN model achieved the highest performance (AUC = 0.8063), followed by the Ensemble (0.7510), Neural Network (0.7351), Decision Tree (0.7204), and SVM (0.6898) models.

The k-NN model was selected as the reference model for statistical comparisons, as it achieved the highest average ROC-AUC among all evaluated models. Pairwise comparisons against this model provide a clearer interpretation of relative performance differences. The results of the DeLong test are summarized in Table 8. The k-NN model significantly outperformed the SVM model (*p* = 0.0287 < 0.05). However, the differences between k-NN and the Decision Tree (*p* = 0.0830), Ensemble (*p* = 0.2592), and Neural Network (*p* = 0.1873) models were not statistically significant at the 0.05 significance level.

These findings indicate that, although the k-NN model achieved the highest average ROC-AUC, its superiority is statistically supported only in comparison with the SVM model. For the remaining model comparisons, no statistically significant differences were observed. This suggests that the observed variations in performance may be attributed to fold-level variability rather than consistent differences in model capability. Accordingly, the results are interpreted in terms of relative performance rather than definitive superiority.

## 4. Discussion

Recent advances in radiomics have provided new opportunities for quantitative tumor characterization and individualized risk prediction in colorectal cancer. Several studies have reported strong associations between CT-derived radiomic features and tumor aggressiveness, perineural invasion, and survival outcomes [20,21]. Consistent with these findings, our study demonstrated that CT-based radiomic features combined with machine learning can effectively differentiate between CEA-positive and CEA-negative colon cancer patients.

Analysis of classification performance across different feature dimensions showed that models trained with 41 selected features achieved the most balanced and stable outcomes. Within this feature space, the k-NN, SVM, and NN classifiers achieved the highest overall accuracy and discriminative power while maintaining an optimal balance between sensitivity and specificity. The SVM and NN models achieved the best recall values, reflecting their superior ability to identify CEA-positive colon cancer cases. This high recall (up to 83.0%) is clinically valuable, as it minimizes the number of false-negative cases—patients who are truly CEA-positive but are missed by the model—which is critical for identifying high-risk individuals requiring stringent postoperative surveillance. These findings indicate that optimal feature selection and algorithmic integration can enhance model generalization and improve predictive consistency across different machine learning approaches. The observed association between radiomic features and serum CEA status suggests that imaging-derived features may provide complementary information regarding tumor biology. Recent studies have suggested that combining imaging-derived quantitative features with biochemical markers may enhance predictive performance beyond either approach alone. For instance, Huang et al. (2018) successfully integrated CT-based radiomic signatures with serum CEA levels to predict perineural invasion in colorectal cancer [22]. Similarly, Lv et al. (2022) demonstrated that combining radiomic features with clinical and serum data improved survival prediction and recurrence risk assessment [23]. In line with these reports, our study highlights that CT-based radiomic modeling can effectively distinguish between CEA-positive and CEA-negative colon cancer patients, suggesting potential for biomarker-enhanced risk stratification. These findings indicate that radiomic features may serve as complementary indicators of tumor biology, strengthening the link between image-based analysis and molecular or serum biomarkers.

In the present study, radiomic features were not combined with serum CEA values within a single predictive model; rather, CT-based radiomic analysis was used to differentiate patients according to their CEA status. Therefore, the findings should be interpreted as reflecting an association between imaging-derived features and biomarker status, rather than a comprehensive integrated model.

In the literature, simpler tree-based or ensemble models have been reported to be more sensitive to redundancy and noise within radiomic datasets, whereas distance- and kernel-based classifiers (such as k-NN and SVM) generally provide higher stability and generalization capacity [24,25,26,27,28]. Consistent with the literature, our study also demonstrated that k-NN, SVM, and NN classifiers achieved consistently superior results compared with other models. In this context, the multi-algorithmic approach and correlation-based feature selection method employed in our study are in line with methodological principles previously recommended in the literature to enhance model robustness [29]. Taken together, these findings emphasize the importance of methodological rigor and appropriate algorithm selection to maximize clinical applicability in radiomics-based predictive modeling.

Recent literature has also emphasized both methodological advances and persistent challenges in radiomics and artificial intelligence research. Topics such as bias in AI systems, the reproducibility of radiomic features, and the role of standardized quality frameworks—such as the Radiomics Quality Score (RQS) and METRICS—have been widely discussed [30]. In our study, a standardized preprocessing workflow and correlation-based feature selection approach were implemented to reduce data redundancy, improve feature stability, and enhance model generalizability. These methodological steps provide significant advantages for achieving reliable and reproducible radiomic analyses.

Although the present study primarily demonstrates a statistical association between radiomic features and CEA status, potential biological explanations may underlie this relationship. Radiomic features are thought to capture intratumoral heterogeneity, which reflects variations in cellular density, necrosis, and stromal composition. More aggressive tumors, often associated with elevated CEA levels, may exhibit increased heterogeneity, irregular vascular architecture, and areas of necrosis, all of which can be reflected in texture-based imaging features. Therefore, radiomic signatures may indirectly represent underlying tumor biology associated with serum biomarker expression, even though they do not directly measure molecular or biochemical processes.

Particularly in oncologic imaging, radiomics has shown considerable potential for tumor characterization and prognostic modeling. Our study extends this perspective by emphasizing that CT-derived radiomic features can complement serum biomarkers such as CEA, contributing to a more comprehensive and personalized approach to tumor assessment and management. Although serum CEA levels can be easily obtained through routine blood testing, imaging-based prediction may provide complementary biological information regarding tumor heterogeneity and aggressiveness. Radiomic features extracted from CT images may reflect intratumoral microstructural characteristics that are not fully captured by circulating biomarkers alone. In this context, radiomics is not intended to replace laboratory testing but rather to provide additional imaging-derived biomarkers that may improve tumor characterization and risk stratification. Therefore, the association between CT-based radiomic signatures and serum CEA status may have potential value in supporting a more comprehensive and biologically informed assessment of colon cancer. From a clinical perspective, the value of imaging-based models may be particularly relevant in cases where serum biomarkers alone provide limited insight into the biological heterogeneity of tumors. Rather than duplicating a laboratory parameter, radiomic analysis may help bridge imaging phenotypes with biochemical tumor characteristics, thereby contributing to multimodal decision-support frameworks in colorectal cancer management.

In the present study, serum CEA levels were found to be significantly higher in smokers, indicating that smoking may act as a confounding factor influencing CEA levels. This finding is consistent with previous reports demonstrating elevated baseline CEA levels in smokers. Therefore, the observed associations between radiomic features and CEA status should be interpreted with consideration of potential confounding effects.

From a clinical perspective, CT-based radiomic models may have potential utility in preoperative risk assessment and decision support. In routine clinical practice, abdominal CT is already widely performed for staging and evaluation of colorectal cancer. Radiomic analysis may provide additional, non-invasive imaging-based information associated with tumor biology and biomarker status, particularly in cases where serum CEA levels are inconclusive or do not fully reflect tumor behavior. Rather than replacing laboratory biomarkers, such models may serve as complementary decision-support tools, contributing to a more comprehensive assessment of patients.

Although the machine learning models demonstrated promising performance in differentiating CEA status, the potential for misclassification should be carefully considered in clinical practice. These models are not intended to function as standalone diagnostic tools but rather as decision-support systems that complement clinical and radiological evaluation. In cases of uncertain or potentially incorrect predictions, re-evaluation of imaging findings and integration with clinical, laboratory, or histopathological data remain essential to ensure accurate diagnosis. Additionally, in the event of misclassification, patients should be informed appropriately with clear explanations and provided with appropriate clinical follow-up to ensure safety. Furthermore, analysis of misclassification patterns may provide valuable insights into model limitations and guide future improvements. Expanding the dataset, refining feature selection strategies, adopting continuous learning pipelines, and optimizing model parameters may further enhance performance. In addition, external validation using independent cohorts and the incorporation of automated segmentation approaches may improve the generalizability and reproducibility of radiomics-based models. Future multicenter studies are warranted to confirm these findings and support the clinical translation of such approaches.

## 5. Conclusions

In conclusion, CT-based radiomic analysis combined with machine learning—particularly k-NN, SVM, and NN classifiers—demonstrated promising performance in differentiating CEA-positive from CEA-negative colon cancer patients. Radiomic features derived from CT images may provide imaging-based information associated with serum CEA status, potentially supporting tumor characterization and contributing to clinical decision-making in colorectal cancer management.

### Limitations

Despite promising results, this study is subject to several limitations that must be addressed. First and foremost, this was a single-center study with a relatively limited sample size (n = 109). Although internal validation using stratified 5-fold cross-validation provided a robust estimate of performance, the findings may not be immediately generalizable to different populations, CT scanners, or acquisition protocols. Furthermore, while we performed a Feature Robustness Analysis to mitigate errors, the reliance on manual segmentation by a single observer for the primary dataset remains a potential source of bias. This bias is compounded by using a limited 3-slice segmentation strategy (a central slice and two adjacent slices), adopted to streamline the manual segmentation workflow, but that may not fully capture the entire 3D tumor volume. Additionally, although internal validation was performed using stratified cross-validation, the absence of an independent external validation cohort limits the generalizability of the model. Although smoking status was analyzed in relation to serum CEA levels, it was not incorporated into the machine learning models, and its potential confounding effect may have influenced the results. Future multicenter studies incorporating external validation datasets are needed to confirm the robustness and clinical applicability of these findings. Future research should focus on multi-center, prospective studies with significantly larger patient cohorts and an independent, external validation set to validate the clinical utility and generalizability of the CEA status prediction model. Automated or semi-automated segmentation methods should also be explored to reduce inter-observer variability and improve workflow efficiency.

## Figures and Tables

**Table 1:** Baseline Clinical and Pathological Characteristics of the Study Population.

Variable	Value
**Age (years)**	55.09 ± 16.81
**Sex, *n* (%)**	
Female	39 (35.8%)
Male	70 (64.2%)
**CEA ≥ 5 ng/mL, *n* (%)**	53 (48.6%)
**TNM Classification, *n* (%)**	
T1N0M0	2 (1.8)
T2N0M0	6 (5.5)
T2N1aM0	5 (4.6)
T3N0M0	35 (32.1)
T3N1aM0	10 (9.2)
T3N2bM0	6 (5.5)
T3N2bM1a	4 (3.7)
T3NOM0	6 (5.5)
T4aN0M1c	5 (4.6)
T4aN2aM0	7 (6.4)
T4aN2bM1	1 (0.9)
T4aN2bM1c	9 (8.3)
T4bN2bM1c	4 (3.7)
TisN0M0	4 (3.7)
TxN2bM1c	5 (4.6)
**Tumor Stage, *n* (%)**	
Stage 0 (Carcinoma in situ)	4 (3.7%)
Stage 1	8 (7.3%)
Stage 2A	41 (37.6%)
Stage 3A	5 (4.6%)
Stage 3B	10 (9.2%)
Stage 3C	14 (12.8%)
Stage 4A	4 (3.7%)
Stage 4B	11 (10.1%)
Stage 4C	12 (11.0%)
**Tumor Type, *n* (%)**	
Adenocarcinoma	105 (96.3)
High-grade dysplastic tubulovillous adenoma with intramucosal adenocarcinoma	4 (3.7)

**Table 2:** Smoking Status and its Association with Serum CEA Levels.

Variable	Value	Mean CEA (ng/mL)	*p*-Value
**Smoking status, *n* (%)**			
Yes	38 (34.9%)	20.78 ± 7.31	
No	71 (65.1%)	4.08 ± 2.56	
**Comparison (Yes vs. No)**			**0.001**

**Table 3:** Multivariate Linear Regression Analysis of Factors Associated with Serum CEA Levels.

Item	Unstandardized Coefficients		*p*-Value	95% Confidence Interval for Beta	
	Beta	Standard Error		Lower Bound	Upper Bound
Smoking	28.589	8.806	0.003	10.673	46.505
Age	−0.260	0.474	0.587	−1.224	0.704
Sex	−3.228	22.961	0.889	−49.942	43.487
Stage	5.202	7.502	0.493	−10.061	20.466
Tumor type	−17.527	42.166	0.680	−103.315	68.261

**Table 4:** Classification performance scores obtained using all 107 features without dimensionality reduction.

Model	Accuracy (%)	Recall (%)	Specificity (%)	F1 Score (%)	Precision (%)	ROC-AUC	Brier Score
**SVM**	75.5 ± 2.0	60.4 ± 2.5	90.6 ± 1.8	71.1 ± 2.1	86.5 ± 1.7	0.7605 ± 0.015	0.219 ± 0.068
**Tree**	70.8 ± 2.2	60.4 ± 2.7	81.1 ± 2.4	67.3 ± 2.3	76.2 ± 1.9	0.7145 ± 0.018	0.365 ± 0.077
**Ensemble**	75.5 ± 1.9	67.9 ± 2.3	83.0 ± 1.9	73.4 ± 1.8	80.0 ± 1.6	0.7727 ± 0.014	0.282 ± 0.059
**k-NN**	79.2 ± 2.0	67.9 ± 2.4	90.6 ± 1.8	76.7 ± 1.9	87.8 ± 1.7	0.8545 ± 0.013	0.168 ± 0.064
**NN**	71.7 ± 2.1	60.4 ± 2.5	83.0 ± 2.1	68.1 ± 2.2	78.0 ± 1.8	0.7460 ± 0.016	0.215 ± 0.081

**Table 5:** Classification performance scores calculated using 22 features obtained after correlation-based feature selection (stratified 5-fold cross-validation, mean ± 95% CI).

Model	Accuracy (%)	Recall (%)	Specificity (%)	F1 Score (%)	Precision (%)	ROC-AUC	Brier Score
**SVM**	72.6 ± 2.1	60.4 ± 2.5	84.9 ± 2.0	68.8 ± 2.3	80.0 ± 1.9	0.7957 ± 0.016	0.216 ± 0.034
**Tree**	66.0 ± 2.3	56.6 ± 2.7	75.5 ± 2.2	62.5 ± 2.4	69.8 ± 2.0	0.6333 ± 0.020	0.241 ± 0.016
**Ensemble**	76.4 ± 2.0	71.7 ±2.4	81.1 ± 1.9	75.3 ±2.1	79.2 ± 1.8	0.7948 ± 0.015	0.273 ± 0.102
**k-NN**	80.2 ± 2.1	69.8 ± 2.3	90.6 ± 1.8	77.8 ± 2.0	88.1 ± 1.6	0.8592 ± 0.013	0.163 ± 0.034
**NN**	79.2 ± 2.0	66.0 ± 2.5	92.5 ± 1.7	76.0 ± 2.1	89.7 ± 1.5	0.7827 ± 0.014	0.186 ± 0.035

**Table 6:** Classification performance scores calculated using 31 features obtained after correlation-based feature selection (stratified 5-fold cross-validation, mean ± 95% confidence interval).

Model	Accuracy (%)	Recall (%)	Specificity (%)	F1 Score (%)	Precision (%)	ROC-AUC	Brier Score
**SVM**	71.7 ± 2.1	56.6 ± 2.5	86.8 ± 1.9	66.7 ± 2.3	81.1 ± 1.7	0.7848 ± 0.016	0.314 ± 0.063
**Tree**	66.0 ± 2.3	62.3 ± 2.6	69.8 ± 2.2	64.7 ± 2.4	67.3 ± 1.9	0.7255 ± 0.018	0.208 ± 0.069
**Ensemble**	61.3 ± 2.4	50.9 ± 2.8	71.7 ± 2.3	56.9 ± 2.5	64.3 ± 2.0	0.6778 ± 0.019	0.211 ± 0.021
**k-NN**	78.3 ± 2.0	69.8 ± 2.4	86.8 ± 1.8	76.3 ± 2.1	84.1 ± 1.6	0.7932 ± 0.014	0.163 ± 0.023
**NN**	73.6 ± 2.1	64.2 ± 2.5	83.0 ± 1.9	70.9 ± 2.2	79.1 ± 1.7	0.7935 ± 0.015	0.213 ± 0.051

**Table 7:** Classification performance scores obtained using 41 features selected with a correlation threshold of 0.7 (stratified 5-fold cross-validation, mean ± 95% confidence interval).

Model	Accuracy (%)	Recall (%)	Specificity (%)	F1 Score (%)	Precision (%)	ROC-AUC	Brier Score
**SVM**	76.4 ± 2.0	83.0 ± 2.3	69.8 ± 2.1	77.9 ± 2.0	73.3 ± 1.8	0.8324 ± 0.014	0.294 ± 0.058
**Tree**	74.5 ± 2.2	67.9 ± 2.5	81.1 ± 2.0	72.7 ± 2.1	78.3 ± 1.9	0.7375 ± 0.017	0.231 ± 0.091
**Ensemble**	74.5 ± 2.1	64.2 ± 2.6	84.9 ± 1.8	71.6 ± 2.0	81.0 ± 1.7	0.7554 ± 0.015	0.495 ± 0.022
**k-NN**	77.4 ± 2.0	79.2 ± 2.3	75.5 ± 1.9	77.8 ± 2.0	76.4 ± 1.8	0.8523 ± 0.013	0.188 ± 0.043
**NN**	78.3 ± 2.0	83.0 ± 2.3	73.6 ± 2.0	79.3 ± 1.9	75.9 ± 1.7	0.8262 ± 0.014	0.309 ± 0.053

**Table 8:** Pairwise comparison of ROC-AUC values using the paired DeLong test based on out-of-fold predictions. * indicates statistically significant differences based on the paired DeLong test (*p* < 0.05).

Reference Model	Compared Model	AUC (Ref)	AUC (Comp)	ΔAUC	Z-Statistic	*p*-Value
**k-NN**	SVM	0.8063	0.6898	0.1166	2.1879	0.0287 *
**k-NN**	Tree	0.8063	0.7204	0.0860	1.7337	0.0830
**k-NN**	Ensemble	0.8063	0.7510	0.0554	1.1284	0.2592
**k-NN**	NeuralNetwork	0.8063	0.7351	0.0712	1.3186	0.1873

## Data Availability

The data presented in this study are available on reasonable request from the corresponding author. The data are not publicly available due to patient privacy and institutional ethical restrictions.

## Abbreviations

The following abbreviations are used in this manuscript:

CEA	Carcinoembryonic Antigen
CT	Computed Tomography
ROC	Receiver Operating Characteristic
AUC	Area Under the Curve
SVM	Support Vector Machine
k-NN	k-Nearest Neighbor
NN	Neural Network
GLCM	Gray Level Co-occurrence Matrix
GLRLM	Gray Level Run Length Matrix
ICC	Intraclass Correlation Coefficient
ROI	Region of Interest
